# Clinical Characteristics of *Clonorchis sinensis*-Associated Cholangiocarcinoma: A Large-Scale, Single-Center Study

**DOI:** 10.3389/fmed.2021.675207

**Published:** 2021-05-28

**Authors:** Jong-In Chang, Keol Lee, Dongwuk Kim, Ju-II Yang, Jae Keun Park, Kyu Choi, Soo Hoon Kang, Kwang Hyuck Lee, Kyu Taek Lee, Jong Kyun Lee, Seon Mee Park, Joo Kyung Park

**Affiliations:** ^1^Division of Gastroenterology, Department of Medicine, Samsung Medical Center, Sungkyunkwan University School of Medicine, Seoul, South Korea; ^2^Division of Gastroenterology, Department of Internal Medicine, Good Gangan Hospital, Busan, South Korea; ^3^Department of Internal Medicine, Kangnam Sacred Heart Hospital, Hallym University College of Medicine, Seoul, South Korea; ^4^Department of Internal Medicine, Chungbuk National University College of Medicine, Cheongju-si, South Korea

**Keywords:** clonorchiasis, *Clonorchis sinensis*, cholangiocarcinoma, endemic, prognosis

## Abstract

**Background:** *Clonorchis sinensis* (CS) infection is considered a group 1 carcinogen of cholangiocarcinoma (CCA). There were very few studies regarding clinical characteristics of CS-associated CCA (CACC). This study aimed to investigate clinical characteristics of patients with CCA with or without CS infection.

**Methods:** A total of 367 patients diagnosed with CCA who underwent diagnostic tests for CS infection were enrolled. CS infection was defined as follows: at least one positive serum ELISA test, skin test, stool microscopy, or bile microscopy.

**Results:** There were 95 (26%) patients with CS infections. The median follow-up duration was 14.9 months (range, 6.07–36.17). The following significant differences were noted among patients with CACC compared to non-CACC; diagnosis at younger age (median 62 years vs. 65 years, *p* = 0.018), higher male to female ratio (83.2 vs. 61.8%, *p* < 0.001), and residence in CS-endemic area (46.3 vs. 25.4%, *p* = 0.014). Univariate analysis of prognostic factors indicated that tumor location, curative resection, tumor stage, and laboratory tests including CA 19-9, CEA, and bilirubin were significantly associated with overall survival, but CS infection was not. In multivariate analysis, tumor location, CEA, curative resection and tumor stage were identified as independent prognostic factors. Among patients under age 64, CACC group had lower survival rate than non-CACC group (*p* = 0.022).

**Conclusions:** CACC had the following significant characteristics compared to non-CACC; diagnosis at younger age, higher male to female ratio, higher prevalence in CS endemic areas and poorer overall survival in patients under age 64.

## Introduction

Clonorchiasis is a parasitic infection by *Clonorchis sinensis* (CS), which people contract by ingestion of metacercariae in raw or undercooked freshwater fish (1). CS infestation can persist in bile duct for at least 26 years and causes various complications in liver and biliary systems, mainly cholelithiasis, cholangitis, and even cholangiocarcinoma (CCA) (2–4). CS infection was considered as a group 1 carcinogen by the International Agency for Research on Cancer (5). In particular, East Asia including Southeast China, Vietnam, and Korea is an endemic area for clonorchiasis, and the estimated CCA incidence rate among the population infected with CS was extremely high, reaching 35 per 100,000 men and 25 per 100,000 women (6).

Recently, the incidence of intrahepatic CCA has increased worldwide, while the incidence of extrahepatic CCA has decreased in Western countries and remained unchanged in Asian countries (3). In East Asia, clonorchiasis is a powerful risk factor along with choledochal cysts, primary sclerosing cholangitis, and hepatolithiasis (7). The proportion of CS-associated CCA (CACC) among entire CCA has ranged widely from 2.2 to 41.4% in previous studies (8–10). Although there is a strong relationship between clonorchiasis and CCA, there have been few studies regarding clinical characteristics and prognosis in patients with CACC.

In this study, we investigated the clinical characteristics and prognosis of CACC compared to CCA without chronic CS infection (non-CACC) among a large number of patients over long-term follow-up.

## Methods

### Patients

This is a retrospective study at Samsung Medical Center, between December 1994 and March 2015. We screened adult patients (over 18 years of age) who met the following inclusion criteria: (1) diagnosed with CCA histologically confirmed by surgical specimen, biopsy or cytology with endoscopic retrograde cholangiopancreatography or endoscopic ultrasound, liver biopsy, bile cytology from percutaneous transhepatic biliary drainage, or core needle biopsy or fine-needle aspiration of lymph nodes (2) underwent diagnostic tests for CS infection before the diagnosis of CCA. A total of 439 patients met the inclusion criteria. Among them, 72 patients who met any of the following criteria were excluded: (1) hepatocellular carcinoma combined with CCA on pathology, (2) a follow-up duration <30 days, or (3) other hepatobiliary malignancies. Therefore, a total of 367 patients were analyzed.

### Definitions and Measurement

Enrolled patients were divided into two groups according to CS infection: the CACC group and the non-CACC group. Evidence of CS infection was defined as a positive result on serologic ELISA or skin test, or microscopic confirmation of CS eggs or adult worms on stool or bile specimens.

We also collected demographic, biochemical, and clinical data including cancer stage, treatment intent, and survival time. The endemic area in this study consisted of the basin of four major rivers (Gum, Seomjin, Youngsan, and Nagdong rivers) located in the southern part of Korea (11). CCA was staged according to the 7th edition of the American Joint Committee on Cancer (AJCC) tumor-node-metastasis staging system. Treatment intent was classified as curative or palliative intent. Curative intent treatment included R0 or R1 resection by segmental common bile duct resection, hepatectomy, or Whipple operation. Palliative intent treatment included R2 resection, repetitive biliary drainage, local radiotherapy, or chemotherapy. Overall survival (OS) was defined as the period between the date of diagnosis of CCA and the date of death or the last day of hospital visitation until November 30, 2015. The data of death were obtained from the national death register.

We evaluated the methodological quality of our study using the Newcastle-Ottawa Scale (NOS) for cohort study (http://www.ohri.ca/programs/clinical_epidemiology/oxford.asp), which is the most frequently used tool nowadays (12, 13).

The study protocol was approved by the Institutional Review Board of Samsung Medical Center. Because this study involved a retrospective analysis of existing clinical data, the need for informed consent from patients was waived (IRB No. 2015-10-129-002).

### Statistics

SPSS 21.0 (IBM, Armonk, NY) was used for statistical analysis. Student's *t*-test, Pearson's chi-square test, Fisher's exact test, and Mann-Whitney *U*-test were used to compare groups with or without CS infection. The Kaplan–Meier method with Log-rank test was performed to compare OS between groups with different clinical parameters. The Cox proportional hazards model was used to identify independent prognostic factors. All tests of significance were two-tailed and a *P* < 0.05 was considered statistically significant.

## Results

### Patient Characteristics

The clinical characteristics of study patients are summarized in Table 1. The median age was 64 years and 67.3% of patients were male. The median follow-up duration was 14.9 months (range: 6.07–36.17). Tumor location was classified as intrahepatic, perihilar, and distal bile duct, which was 125 (34.1%), 128 (34.9%), and 114 (31.1%), respectively. Eighty-seven patients (23.7%) were stage I, 50 (13.6%) were stage II, 85 (22.9%) were stage III, and 146 (39.8%) were stage IV. Among them, 170 (46.3%) patients were treated with curative intent, while 196 (53.6%) were treated with palliative intent.

**Table 1:** Baseline characteristics of 367 cholangiocarcinoma study patients.

**Characteristics**	**Value, *n* (% or range)**
Age (years)	64 (58–70)
Gender, male	247 (67.3)
Follow-up duration, months	14.9 (6.07–36.17)
Diabetes mellitus	54 (14.7)
Obesity (BMI ≥ 29)	8 (2.2)
HBV infection	26 (7.1)
HCV infection	3 (0.8)
CA 19-9 (U/ml)	148.1 (32.9–1467.4)
CEA (U/ml)	86.8 (1–11029)
**Tumor location**	
Intrahepatic	125 (34.1)
Perihilar	128 (34.9)
Distal	114 (31.1)
**Cancer stage by AJCC, 7th ed**.	
I (IA, IB)	87 (23.7)
II (IIA, IIB)	50 (13.6)
III (IIIA, IIIB)	85 (22.9)
IV (IVA, IVB)	146 (39.8)
**Treatment intent**	
Curative intent (R0, R1 resection)	170 (46.3)
Palliative treatment^*^	196 (53.6)
CS infection (+)	95 (25.9)

*CS, Clonorchis sinensis; CCA, cholangiocarcinoma; CEA, carcinoembryonic antigen; CA 19-9, Carbohydrate antigen 19-9; AJCC, American Joint Committee on Cancer*.

*Data are expressed as n (%) or median (quartile range) or mean ± SD*.

* *Palliative treatment included R2 resection, radiation therapy, chemotherapy, or only supportive care*.

### Comparison of Clinical Characteristics According to CS Infection

The CACC group and the non-CACC group included 95 (25.9%) and 272 (74.1%) patients, respectively. The proportion of CACC and non-CACC did not change over 20 years, with a proportion of CACC during the two periods, 1995–2004 and 2005–2015, of 25.6% and 25.7% (*p* = 0.021).

Comparison of clinical characteristics of the two groups is summarized in Table 2. The CACC group was significantly younger at diagnosis of CCA (median, 65 years vs. 62 years, *p* = 0.018), had higher male to female ratio (83.2 vs. 61.8%, *p* < 0.001), more frequent elevated serum CEA levels (>5 U/ml), and included more patients living in CS-endemic areas (46.3 vs. 25.4%, *p* = 0.014) than the non-CACC group. The non-CACC group had more patients with a history of hepatolithiasis than the CACC group (12.9 vs. 3.2%, *p* = 0.005).

**Table 2:** Comparison of clinical characteristics of cholangiocarcinoma patients according to *Clonorchis sinensis* infection.

**Characteristics**	**CS (+) CCA (*n* =95)**	**CS (-) CCA (*n* = 272)**	***p*-value**
Age (years)	62 (56–68)	65 (58–70)	0.018
Gender, male	79 (83.2)	168 (61.8)	<0.001
Endemic area	44 (46.3)	69 (25.4)	0.014
Diabetes mellitus	9 (9.5)	45 (16.5)	0.09
BMI (kg/m^2^)	22.8 ± 2.89	22.7 ± 2.63	0.61
HBV infection	5 (5.3)	21 (7.7)	0.42
Hepatolithiasis	3 (3.2)	35 (12.9)	0.006
CA 19-9 (>37 U/ml)	60/91 (65.9)	202/266 (75.9)	0.074
CEA (>5 U/ml)	21/67 (31.3)	31/183 (16.9)	0.021
**Tumor location**			0.39
Intrahepatic	32 (33.7)	93 (34.2)	
Perihilar	38 (40.0)	90 (33.1)	
Distal	25 (26.3)	89 (32.7)	
**Cancer stage by AJCC, 7th ed**.			
I, II	30 (31.6)	107 (39.5)	0.36
III, IV	65 (68.4)	165 (60.5)	
**Treatment intent**			
Curative intent	38 (40.0)	132 (48.7)	0.14
Palliative treatment^*^	57 (60.0)	139 (51.3)	
Tumor differentiation^**^			
Well/moderate	38 (73.1)	112 (68.5)	0.21
Poorly/unknown	14 (26.9)	51 (31.3)	

*CS, Clonorchis sinensis; CCA, cholangiocarcinoma; CEA, carcinoembryonic antigen; CA 19-9, Carbohydrate antigen 19-9; AJCC, American Joint Committee on Cancer*.

*Data are expressed as n (%) or median (quartile range) or mean ± SD*.

* *Palliative treatment included R2 resection, radiation therapy, chemotherapy, or only supportive care*.

** *Tumor differentiation was evaluated in 215 patients in whom pathologic analysis was performed*.

### Survival According to CS Infection

**Figure 1:**
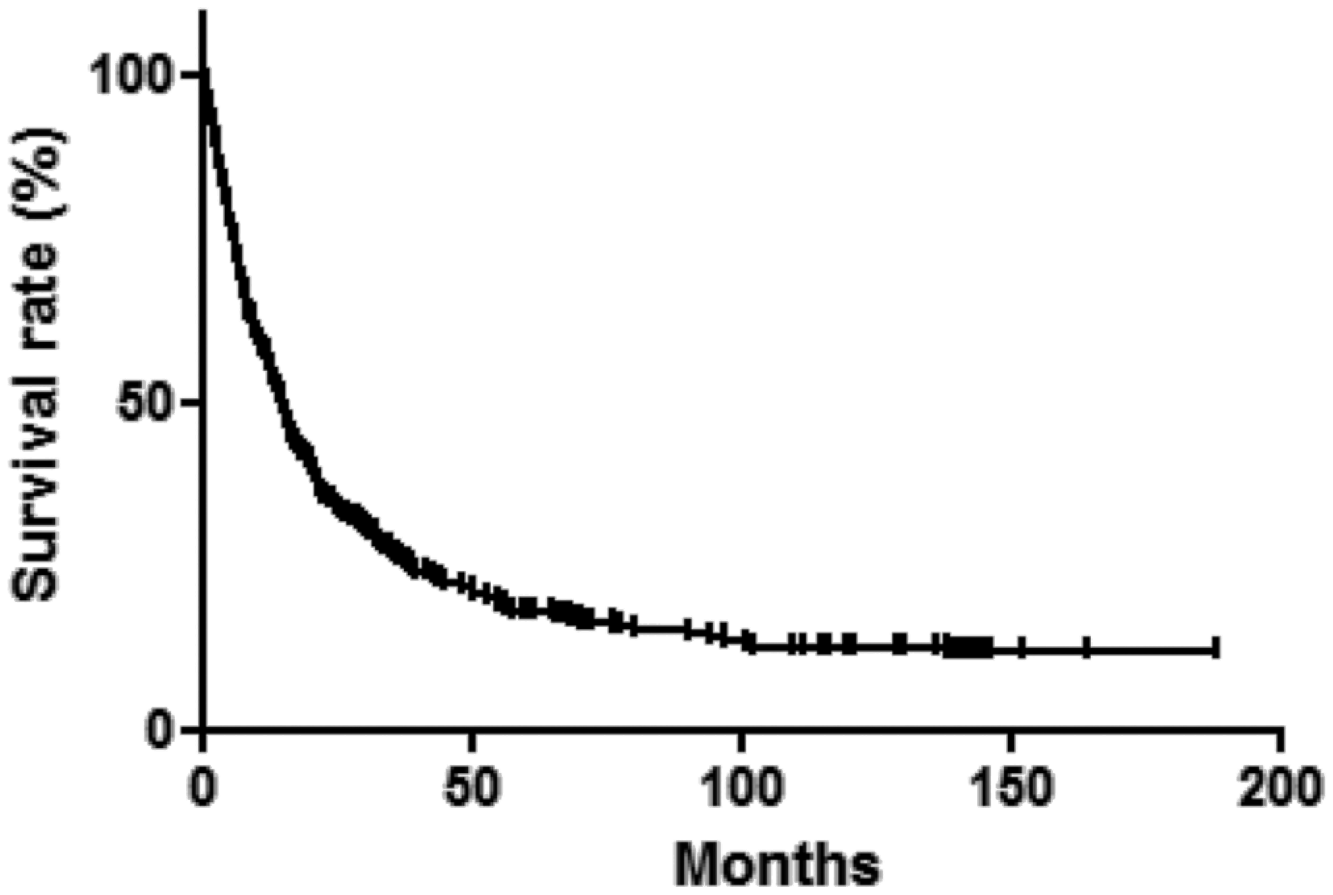
Cumulative survival rate in study patients (1-year: 57.5%, 3-year: 27.2%, 5-year: 18.2%).

**Figure 2:**
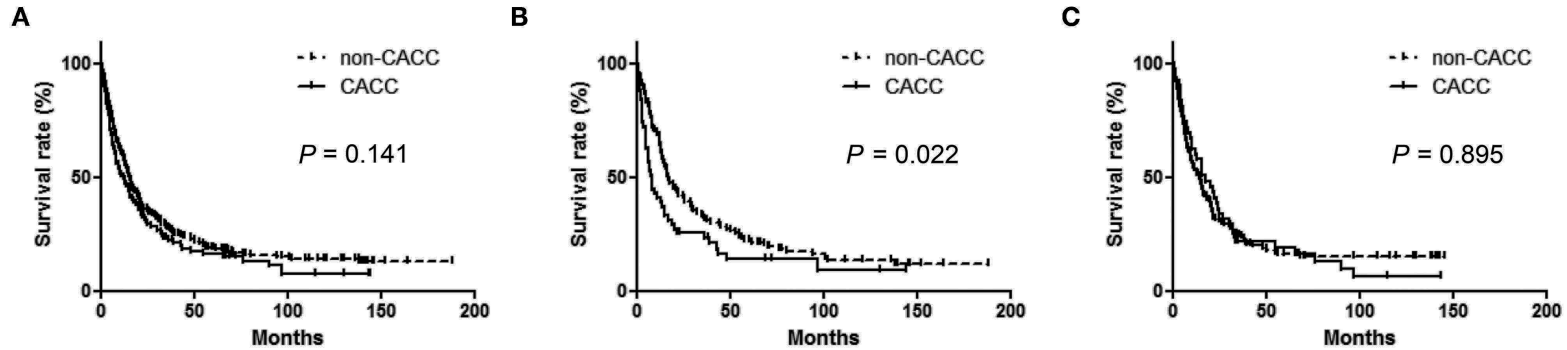
Kaplan-Meier curves according to *Clonorchis sinensis* infection in analysis: **(A)** The entire cohort **(B)** Age <64 years **(C)** Age ≥ 64 years. Comparison of median survival time in patients younger than 64 years old between *Clonorchis sinensis*-associated cholangiocarcinoma (CACC) and non-*Clonorchis sinensis*-associated cholangiocarcinoma (non-CACC).

Among the 367 CCA patients, 308 died during the follow-up period. 1-, 3-, and 5-year survival was 57.9, 27.2, 18.2%, respectively, with a median OS of 15.3 months (95% CI, 13.3–17.3) (Figure 1). The survival rate of the CACC group was not significantly different from that of the non-CACC group, with a median OS of 12.7 months (95% CI, 7.56–17.84) and 15.7 months (95% CI, 13.5–17.9), respectively (*p* = 0.141) (Figure 2A). Subgroup analysis divided according to median age of 64 years indicated that the CACC group had a significantly lower survival rate than the non-CACC group, with median OS of 8.2 months (95% CI 5.2–11.2) and 17.1 months (95% CI 11.8–22.4), respectively, in patients aged under 64 years (*p* = 0.022) (Figure 2B); however, OS was similar among those aged 64 or over regardless of CS infection (Figure 2C).

Among patients aged under 64 years, elevated CEA levels (>5 U/ml) (34.3 vs. 13.0%, *p* = 0.019) and smoking history (55.6 vs. 27.1%, *p* = 0.004) were more frequent in the CACC group compared to the non-CACC group. Among patients aged 64 years or over, the characteristics of the CACC group and non-CACC group were similar. Subgroup analysis according to sex and the endemic area did not show survival differences between younger and older groups.

### Prognostic Factors for CCA

In univariate analysis, tumor location, cancer stage, treatment intent, CA 19-9, CEA, and total bilirubin were significantly associated with overall survival in CCA patients (Table 3). To identify independent prognostic factors for survival, all parameters under a *P*-value of 0.2 in univariate analysis were included in multivariable Cox proportional hazards regression analysis. Tumor location, CEA levels, cancer stage, and treatment intent were independent prognostic factors in CCA patients (Table 4). We had a subgroup analysis for the patients under 64 years old. Univariate analysis showed CS infection, tumor location, cancer stage, tumor differentiation, treatment intent, CA 19-9, CEA, and total bilirubin are associated with overall survival in CCA patients under 64 years old. In multivariate analysis, treatment intent and cancer stage were independent prognostic factors in CCA patients under 64 years old.

**Table 3:** Univariate analyses of prognostic factors associated with overall survival in 367 cholangiocarcinoma patients.

**Variables**	**Median OS (95% CI)**	***p*-value**
**CS infection**		
Yes	12.7 (7.56–17.84)	0.141
No	15.7 (13.46–17.95)	
**Age (years)**		
<64	15.1 (12.18–18.04)	0.580
≥64	15.3 (12.35–18.31)	
**Sex**		
Male	15.5 (11.47–19.59)	0.303
Female	13.8 (10.26–17.27)	
**Diabetes mellitus**		
Yes	14.9 (11.69–18.24)	0.883
No	15.5 (12.93–18.00)	
**Hepatolithiasis**		
Yes	8.07 (4.79–11.34)	0.445
No	15.53 (1,301–18.06)	
**Tumor location**		
Intrahepatic	8.1 (6.41–9.72)	<0.001
Perihilar	16.4 (12.77–19.9)	
Distal bile duct	21.4 (12.25–30.56)	
CEA^**^		
≤ 5 ng/ml	19.9 (16.51–23.42)	<0.001
>5 ng/ml	5.6 (2.54–8.59)	
**CA 19-9**		
≤ 37 U/ml	26.9 (14.19–39.61)	<0.001
>37 U/ml	12.8 (10.54–15.16)	
**Total bilirubin**		
≤ 10 mg/dL	15.9 (13.12–18.75)	<0.013
>10 mg/dL	12.5 (7.86–17.21)	
**Treatment intent**		
Curative intent (R0, R1 resection)	36.2 (28.51–43.82)	<0.001
Palliative treatment^*^	7.4 (5.88–8.86)	
**Cancer stage by AJCC, 7th ed**.		
I (I, IA, IB)	65.4 (23.30–107.47)	<0.001
II (II, IIA, IIB)	25.4 (13.04–37.76)	
III (III, IIIA, IIIB)	15.2 (11.71–18.62)	
IV (IV, IVA, IVB)	7.3 (5.98–8.56)	
Tumor differentiation^***^		
Well/moderate	32.6 (22.17–43.09)	<0.001
Poorly/unknown	12.7 (9.90–15.56)	

*OS, overall survival; CS, Clonorchis sinensis; CCA, cholangiocarcinoma; CEA, carcinoembryonic antigen; CA 19-9, Carbohydrate antigen 19-9; AJCC, American Joint Committee on Cancer*.

* *Palliative treatment included R2 resection, radiation therapy, chemotherapy, or only supportive care*.

** *CEA data were available for 250 patients*.

*** *Tumor differentiation was evaluated in 215 patients in whom pathologic analysis was performed*.

**Table 4:** Multivariate analysis using the Cox proportional hazards M = model in cholangiocarcinoma patients.

**Variables**	**Hazard ratio (95% CI)**	***p*-value**
**Tumor location**		0.010
Intrahepatic	1	
Perihilar	1.088 (0.751–1.577)	
Distal	1.957 (1.212–3.162)	
CEA^**^		<0.001
≤ 5 ng/ml	1	
>5 ng/ml	2.351 (1.622–3.407)	
**Treatment for cancer**		<0.001
Curative intent (R0, R1 resection)	1	
Palliative treatment^*^	2.940 (2.026–4.268)	
**Cancer stage by AJCC, 7th ed**.		<0.001
I (I, IA, IB)	1	
II (II, IIA, IIB)	2.317 (1.258–4.268)	
III (III, IIIA, IIIB)	2.481 (1.539–3.999)	
IV (IV, IVA, IVB)	4.766 (2.725–8.335)	

*OS, overall survival; CS, Clonorchis sinensis; CCA, cholangiocarcinoma; CEA, carcinoembryonic antigen; AJCC, American Joint Committee on Cancer*.

* *Palliative treatment included R2 resection, radiation therapy, chemotherapy, or only supportive care*.

** *CEA data were available for 250 patients*.

## Discussion

This hospital-based retrospective cohort study demonstrated that clonorchiasis is still a main cause of CCA. The total proportion of CACC was 25.9% but waSubgroup analysis divided accordings 46.3% in the endemic area. Patients with CACC had different characteristics compared to those with non-CACC: younger age at diagnosis of CCA, higher male to female ratio, higher prevalence in CS-endemic areas, and poorer overall survival in patients aged under 64 years.

The prevalence of clonorchiasis, the most common parasite in Korea, decreased from 2.42% in 2004 to 1.86% in 2012 among the general population (8, 14). Infection rates of CS have also decreased in endemic areas along major river basins (11). Despite the decline in clonorchiasis prevalence, the proportion of CACC was still high and not decreased in the recent 10 years compared to the previous 10 years in this study. The reason for this difference in CS infection rate and CACC proportion remains unclear. We suggest that CCA developed due to irreversible pathogenic pathway despite CS eradication. In a recent study of China, the duration of raw fish consumption more than 28 years was related to tumorigenesis in patients with clonorchiasis (15). We expect that CS-associated disease will continue to be a major public health concern in Korea for a while even if raw fish consumption decreases.

The proportions of CACC in this study were higher than those of previous studies in Korea. A prior study reported that CS infestation causes one-fourth of CCA in the endemic area and approximately 10% of CCA among the general population (3). The differences might have been caused by variation in the diagnostic criteria of CACC. The proportion of CACC varies on the study population and diagnostic criteria. It was from 2.2 to 4.2% when diagnosed by direct stool microscopy and 41.4% using any positive test among stool exam, ELISA test, skin test, bile or surgical specimens, or imaging criteria (8–10). Microscopic examination of the feces for eggs is the gold standard for the diagnosis of CS infection. However, stool exam cannot diagnose past infections and is less sensitive to mild infections (16). Compared to stool exam, ELISA test is an accurate diagnostic method with sensitivity up to 92.5% and specificity up to 93.1% (17). However, ELISA test showed low sensitivity for past CS infection because of low serologic titers (18). Imaging diagnosis by ultrasonography and CT are auxiliary methods for the diagnosis of CS infection (19). Imaging tests are important for the diagnosis of past infections with identifying bile duct changes. Until accurate diagnostic methods are developed, we recommend performing all tests because these tests are specific but less sensitive.

The median age of the patients at the time of diagnosis with CACC was younger than non-CACC. This finding is compatible with previous results that CCA develops earlier in patients with risk factors such as choledochal cysts at a median age of 42 years or primary sclerosing cholangitis in their 40 s (20, 21). Persistent chronic inflammatory responses may lead to malignant transformation at earlier time points.

Seventy percentage of CCAs are sporadic and 30% are associated with more than one risk factor, such as primary sclerosing cholangitis, hepatolithiasis, Caroli's disease, hepatitis B or C infection, liver flukes, cirrhosis, diabetes, obesity, or alcohol (22). Risk factors related to CCA vary by location. In East Asia, CS infection is the most common risk factor, especially in CS-endemic areas. The causative role of CS in intrahepatic CCA has been established (15). However, the role of CS in hilar or extrahepatic CCA has been defined in only a few studies (8). In this study, the proportions of hilar and distal CACC were similar to intrahepatic CCA. CS infection is related to extrahepatic CCA as well as intrahepatic CCA.

In this cohort, hepatitis B or C, and hepatolithiasis were identified in 7.1, 0.8, and 10.4% of patients. Hepatitis B and hepatolithiasis were more prevalent in intrahepatic CCA, which indicated an etiologic role in intrahepatic CCA. HCV infection was very rare, consistent with previous studies in Koreans and other Asian populations (9, 10, 23). Clonorchiasis is a risk factor of hepatolithiasis and silent CCA develops in 10% of patients with hepatolithiasis even after removal of stones (24). CS causes recurrent pyogenic cholangitis as a nidus for stone formation or by damaging the bile ducts, resulting in stricture and stone formation (25). However, a positive immunodiagnosis of clonorchiasis in hepatolithiasis patients was only seen in 6.9% of patients in a Taiwanese study, although this was higher compared to that in controls (0.8%) (26). This study revealed that 10% of CCA patients had hepatolithiasis and 7.9% had clonorchiasis. However, the rates of hepatolithiasis in CACC and non-CACC were 3.2 and 12.9%, respectively. We suggest that clonorchiasis and hepatolithiasis are independent risk factors of CCA.

CACC had a poor prognosis compared to non-CACC in the group <64 years old. However, multivariate analysis revealed prognostic factors are not CS infection but stages, curative resection, tumor location, and CEA levels. Tumor location has been reported as an important prognostic factor of CCA. In this study, distal CCA had a survival advantage over hilar or intrahepatic CCA. The previous studies showed the difference in the prognosis of CCA with three locations and survival advantage in distal CCA (27). Also, high CEA levels were related to poor prognosis, as shown in previous studies. Preoperative CEA levels were related to tumor stage survival rate and tumor recurrence in hilar and intrahepatic CCA (28–30). Earlier stage, curative resection, and low serum CEA levels were favorable prognostic factors in a previous study (15). High CA19-9 levels (>37 U/ml) and hyperbilirubinemia (>10 mg/dL) were poor prognostic factors in CCA on univariate analysis but not on multivariate analysis. The prognostic roles of these factors have been reported in previous studies. Pre-treatment low CA19-9 levels (≤ 1,000 U/ml) and a decline of more than 50% after treatment were significant for predicting a favorable prognosis (9).

Outcomes were poor in CACC patients under 64 years of age at diagnosis in univariate analysis. The reason for poor prognosis in CACC compared with non-CACC in younger patients is not clear. Younger CACC patients had a more frequent smoking history and higher CEA levels. Smoking is a risk factor for intrahepatic and hilar CCA (9). Because high serum CEA level is a poor prognostic factor (31), CACC patients showed a poorer prognosis than non-CACC patients.

This study has some potential limitations. First, all patients did not undergo all tests for CS infection. Therefore, it is possible to underestimate CS infection. Secondly, we compared regional differences in the CS infection rate between endemic areas and non-endemic areas using living area data. Because CS resides in the biliary tree with a long-term period, residence data for childhood and adulthood are also important. Since this is a retrospective study, this information could not be obtained. Thirdly, this study was conducted at a single tertiary center retrospectively. There was the possibility of selection bias. Nevertheless, the results of this study are meaningful because this study included many patients with CACC who underwent several diagnostic tests for CS infection and had sufficient follow-up duration.

In conclusion, this study demonstrated that the clinical characteristics and prognosis of CACC compared to non-CACC. CACC was associated with early-onset, male predominance, endemic area residence, and poor survival in those under 64 years of age compared to non-CACC. The rates of CS infection have decreased in Korea; however, the incidence of CACC has not changed. Large multicenter prospective studies are needed to clarify the characteristics of CACC and the development of targeted therapies that will reverse or block CS-induced chronic inflammatory responses (32).

## Data Availability Statement

The raw data supporting the conclusions of this article will be made available by the authors, without undue reservation.

## Ethics Statement

The studies involving human participants were reviewed and approved by Institutional Review Board of Samsung Medical Center (IRB No. 2015-10-129-002). Written informed consent for participation was not required for this study in accordance with the national legislation and the institutional requirements.

## Author Contributions

JP, JL, and SP: conceptualization (ideas, formulation or evolution of overarching research goals and aims). JP, KeL, J-IY, and SK: data curation. JP, KeL, and SP: formal analysis. JP and SP: funding acquisition. KwL, KyL, and JL: Investigation. JP, KwL, KyL, and JL: methodology. KyL and JL: project administration. KwL, KyL, and JL: resources. JP, KwL, JL, and SP: supervision. JP, KyL, JL, and SP: validation. KeL, SK, and J-IY: visualization. KeL, DK, and J-IC: writing original draft. JP, SP, KC, DK, and J-IC: writing, review, and editing. All authors contributed to the article and approved the submitted version.

## Conflict of Interest

The authors declare that the research was conducted in the absence of any commercial or financial relationships that could be construed as a potential conflict of interest.

## Abbreviations

CS: *Clonorchis sinensis*
CCA: cholangiocarcinoma
CACC: *Clonorchis sinensis*-associated CCA
CEA: carcinoembryonic antigen
CA 19-9: carbohydrate antigen 19-9
CT: computed tomography
ELISA: enzyme-linked immunosorbent assay
AJCC: American Joint Committee on Cancer
OS: overall survival.
