# Supplementary material for: Clinical Characteristics of Clonorchis sinensis-Associated Cholangiocarcinoma: A Large-Scale, Single-Center Study
Source: Front Med (Lausanne). 2021 May 28;8:675207. doi: 10.3389/fmed.2021.675207 (PMC8193222; doi:10.3389/fmed.2021.675207)
Supplement: Supplementary file 1 [file Data_Sheet_1.docx]

**Supplementary Table 1** Diagnostic methods of cholangiocarcinoma

|  | Intrahepatic CCA  (n=125) | Perihilar CCA  (n=128) | Extrahepatic CCA  (n=114) |
| --- | --- | --- | --- |
| Methods |  |  |  |
| Surgery | 41 | 57 | 86 |
| ERCP/EUS | 12 | 31 | 16 |
| Liver biopsy | 52 | 7 | 7 |
| Others* | 20 | 33 | 5 |

*CCA* cholangiocarcinoma *, ERCP* endoscopic retrograde cholangiopancreatography, EUS endoscopic ultrasound

* Others included cytology of malignant ascites, bile cytology from percutaneous transhepatic biliary drainage, or core needle biopsy/fine needle aspiration of lymph nodes.

**Supplementary Table 2** Prognostic factors associated with overall survival in patients <64 yeas old (n=169)

| Variables | **Univariate analysis** | | **Multivariate analysis** | |
| --- | --- | --- | --- | --- |
|  | Hazard ratio (95% CI) | *p*-value | Hazard ratio (95% CI) | *p*-value |
| CS infection (yes) | 1.51 (1.06 – 2.16) | 0.027 | 1.02 (0.52 – 2.03) | 0.48 |
| Male sex (vs female) | 1.05 (0.74 – 1.49) | 0.780 |  |  |
| Diabetes mellitus (yes) | 0.68 (0.39 – 1.18) | 0.169 | 0.81 (0.30 – 2.1) | 0.675 |
| Hepatolithiasis (yes) | 1.20 (0.69 – 2.09) | 0.520 |  |  |
| Tumor location  Intrahepatic  Perihilar  Distal bile duct | Reference  0.65 (0.44 – 0.95)  0.54 (0.35 – 0.82) | 0.026  0.004 | Reference  1.15 (0.41 – 3.19)  1.45 (0.45 – 4.62) | 0.795  0.532 |
| CEA^**^  ≤ 5 ng/ml  > 5 ng/ml | Reference  2.33 (1.44 – 3.79) | 0.001 | Reference  1.78 (0.83 – 3.84) | 0.141 |
| CA 19-9  ≤ 37 U/ml  > 37 U/ml | Reference  1.85 (1.23 – 2.80) | 0.003 | Reference  1.08 (0.56 – 2.10) | 0.815 |
| Total bilirubin  ≤ 10 mg/dL  > 10 mg/dL | Reference  1.70 (1.16 – 2.51) | 0.007 | Reference  2.48 (0.83 – 7.36) | 0.103 |
| Treatment intent  Curative intent (R0, R1 resection)  Palliative treatment^*^ | Reference  4.29 (2.95 – 6.22) | <0.001 | Reference  2.30 (1.15 – 4.60) | 0.019 |
| Cancer stage by AJCC, 7th ed.  I (I, IA, IB)  II (II, IIA, IIB)  III (III, IIIA, IIIB)  IV (IV, IVA, IVB) | Reference  2.89 (1.44 – 5.81)  4.87 (2.53 – 9.00)  10.55 (5.88 – 18.93) | 0.003  <0.001  <0.001 | Reference  1.93 (0.52 – 7.10)  2.39 (0.75 – 7.61)  6.28 (1.67 – 23.54) | 0.324  0.140  0.006 |
| Tumor differentiation^***^  Well/moderate  Poorly/unknown | Reference  1.68 (1.07 – 2.66) | 0.026 | Reference  1.44 (0.76 – 2.75) | 0.268 |

*OS* overall survival, *CS* *Clonorchis sinensis,* *CCA* cholangiocarcinoma, *CEA* carcinoembryonic antigen, *CA 19-9* Carbohydrate antigen 19-9, *AJCC* American Joint Committee on Cancer

^*^Palliative treatment included R2 resection, radiation therapy, chemotherapy, or only supportive care.

^**^CEA data were available for 112 out of 169 patients.

^***^Tumor differentiation was evaluated in 105 out of 169 patients in whom pathologic analysis was performed.
